# Effective Modeling of Continuous Wave Z‐Spectra for Quantitative CEST and CESL MRI Across Exchange Regimes

**DOI:** 10.1002/mrm.70483

**Published:** 2026-07-30

**Authors:** Chris Lippe, Verena Hoerr

**Affiliations:** ^1^ Multiscale Imaging Centre, Clinic of Radiology University of Münster Münster Germany; ^2^ Heart Center Bonn, Department of Internal Medicine II University Hospital Bonn Bonn Germany

**Keywords:** Bloch‐McConnell equations, CEST, quantitative MRI, spin‐lock

## Abstract

**Purpose:**

Chemical exchange saturation transfer (CEST) and chemical exchange‐sensitive spin‐lock (CESL) MRI enable spectroscopic imaging with high sensitivity, but conventional analysis methods often fail as exchange approaches the fast‐exchange limit. We introduce the AB‐MT+n model for robust, reproducible quantification of continuous‐wave Z‐spectra across exchange regimes.

**Theory and Methods:**

The AB‐MT+n model describes continuous‐wave Z‐spectra by a five‐parameter basis for direct water saturation, semi‐solid magnetization transfer, and fast exchange, which can be extended by off‐resonant components for separable slow‐ and intermediate‐exchanging pools. Performance was evaluated in simulations, phantoms, and in vivo glucose‐enhanced CESL (glucoCESL) of a mouse brain.

**Results:**

Simulations showed that AB‐MT+n recovers the maximally accessible information from Z‐spectra with minimal parameterization, with lower model orders sufficing for faster exchange. In phantoms, the slower‐exchanging glutamate pool was quantified reliably by Lorentzian analysis ($R^{2}>95\%$), whereas fast‐exchanging glucose was not captured accurately by Lorentzian, MTR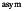, or the full analytical model under varying glutamate concentration ($R^{2}<45\%$). In contrast, the pool‐unspecific asymmetry parameter A preserved strong linearity with glucose concentration ($R^{2}>80\%$). Reduced model orders also sufficed in seven‐pool gray matter simulations and enabled robust quantification of in vivo glucoCESL data.

**Conclusion:**

AB‐MT+n provides an accurate, acquisition‐independent, modular description of continuous‐wave Z‐spectra and improves quantification, particularly in the fast‐exchange regime.

## 1 Introduction

Chemical exchange saturation transfer (CEST) MRI [1] and chemical exchange‐sensitive spin‐lock (CESL) MRI [2] are powerful spectroscopic imaging techniques that leverage chemical exchange of labile protons for contrast enhancement. Studies have demonstrated a wide range of potential applications in which chemical exchange (CE) allows for detection of metabolites at millimolar concentration [3, 4]. Nevertheless, various challenges regarding the quantification of CEST/CESL MRI data persist, including long acquisition times, motion artifacts, sensitivity to $B_{0}$‐, $B_{1}$‐ and $T_{1}$‐inhomogeneities, and low spectral resolution/selectivity  [5, 6].

In general, two different quantification paradigms for CEST/CESL MRI data exist: metric‐based [7] and model‐based [8] analysis. Often employed metrics are magnetization transfer ratio asymmetry

(1) $${\text{MTR}}_{\text{asym}}=Z(-\Delta \omega )-Z(+\Delta \omega ),$$

as well as magnetization transfer ratio $R_{\text{ex}}$

(2) $${\text{MTR}}_{\text{Rex}}=1/Z_{\text{lab}}-1/Z_{\text{ref}},$$

and apparent exchange‐dependent relaxation

(3) $$\text{AREX}={\text{MTR}}_{\text{Rex}}/T_{1},$$

which require a labeled Z value $Z_{\text{lab}}$ that includes exchange effects and one reference measurement $Z_{\text{ref}}$ without exchange effects. For ${\text{MTR}}_{\text{asym}}$, the reference signal is approximated using the signal at the opposite frequency offset. AREX additionally requires $T_{1}$ to be provided.

These metrics are usually easy‐to‐calculate and robust, but have limited capabilities of correcting for field inhomogeneities and separation of individual CE effects. Model‐based approaches have potential to overcome these limitations and also allow for extracting of physically meaningful quantities like proton fraction, exchange and relaxation rates. However, for in vivo Z‐spectroscopy, models often become very complex due to the large amount of involved exchanging pools, impairing stability and reproducibility of fit results. This work aims to investigate and develop further existing model‐based analysis techniques for CEST/CESL MRI towards robust and reproducible quantification of Z‐spectra, with a particular focus on fast‐exchanging pools.

All models used for Z‐spectra quantification originate from Bloch‐McConnell (BM) equations [9], which extend Bloch equations by the exchange of magnetization between multiple pools, for example through CE of labile protons. BM equations can be numerically solved for a series of irradiation offsets Δω to predict the Z‐spectrum of a multi‐pool system. Alternatively, analytical solutions exist that require approximations to different amounts. The most general model we consider here is the approximate solution for the Z‐spectrum under continuous wave (cw) irradiation proposed by Zaiss et al. [10, 11], based on the work of Trott and Palmer [12, 13]. Although, cw irradiation is not applicable in clinical settings due to specific absorption rate (SAR) restrictions, models originally derived under cw assumptions are widely used also on human CEST MRI data [14, 15]. Also, there are large efforts made to restore cw spectra properties using pulsed saturation schemes [16].

This work introduces the AB‐MT+n model, which transforms the analytical model from Zaiss et al. into a modular expression of a five parameter basis set to describe global effects of direct water saturation (DS), magnetization transfer (MT) of semi‐solid proteins and fast‐exchanging pools, extensible for sharp‐resonant effects of slow‐ to intermediate‐exchanging pools. First, we demonstrated that the AB‐MT+n model can recover the maximally accessible information from Z‐spectra in a simulated four‐pool toy‐model, and we investigated how this recoverable information depends on the simulation parameters. Second, we systematically compared the quantification performance of different models, parameters, and metrics, including AB‐MT+n, in dedicated phantom experiments. Finally, we demonstrated the applicability of the AB‐MT+n model under in vivo‐like conditions using a simulated gray matter (GM) system and measured glucose enhanced CESL (glucoCESL) data from a mouse brain.

## 2 Theory

### 2.1 Analytical Solutions to Bloch‐McConnell Equations for Quantitative Z‐Spectroscopy

For long saturation times $t_{\text{sat}}>>T_{2}$, and sufficiently strong saturation $\omega_{1}=\gamma \cdot B_{1}>>1/T_{2}$, BM equations are approximately solved by a one‐dimensional, mono‐exponential decay along the z‐axis [10, 17]. Specifically, the normalized z‐magnetization, that is, the Z‐spectrum, is given by

(4) $$Z(\Delta \omega ,t_{\text{sat}})=\left(P_{z,\text{eff}}P_{z}Z_{i}-Z_{ss}(\Delta \omega )\right)\;e^{-R_{1\rho }(\Delta \omega )\;t_{\text{sat}}}+Z_{ss}(\Delta \omega ),$$

where $Z_{i}=|M_{i}|/M_{0}$ is the initial (normalized) magnetization, Δω is the frequency offset from the water resonance, $P_{z,\text{eff}}$ and $P_{z}$ are projection factors ($P_{z,\text{eff}}=P_{z}=1$ for CESL and $P_{z,\text{eff}}=P_{z}=\cos\theta$ for CEST), $Z_{ss}(\Delta \omega )$ is the steady‐state magnetization achieved for long $t_{\text{sat}}$. The steady‐state $Z_{ss}$ is given by

(5) $$Z_{ss}(\Delta \omega )=\frac{P_{z}\;R_{1,\text{res}}(\Delta \omega )}{R_{1\rho }(\Delta \omega )},$$

where the residual relaxation rate in the rotating frame $R_{1,\text{res}}$ is related to the intrinsic longitudinal relaxation rate of water $R_{1w}$ by

(6) $$R_{1,\text{res}}(\Delta \omega )\approx R_{1w}\cdot \cos\theta \;,\;\text{with}\;\theta =\tan^{-1}(\frac{\omega_{1}}{\Delta \omega }).$$

Furthermore, the rate $R_{1\rho }(\Delta \omega )$ can be decomposed into:

(7) $$R_{1\rho }(\Delta \omega )=R_{\text{eff}}(\Delta \omega )+\underset{i}{\sum }R_{\text{ex},i}(\Delta \omega ),$$

where $R_{\text{eff}}$ describes the intrinsic (direct) water relaxation under RF irradiation:

(8) $$R_{\text{eff}}(\Delta \omega )=\frac{R_{1w}\cdot \Delta \omega^{2}+R_{2w}\cdot \omega_{1}^{2}}{\Delta \omega^{2}+\omega_{1}^{2}},$$

while the exchange contribution $R_{\text{ex},i}$ for each pool is given by:

(9) $$R_{\text{ex},i}(\Delta \omega )=\frac{f_{i}\cdot \omega_{1}^{2}}{\Gamma_{i}^{2}⁄4+{\left(\Delta \omega -\delta \omega_{i}\right)}^{2}}\cdot (R_{2i}+k_{i}\frac{\delta \omega_{i}^{2}+R_{2i}(R_{2i}+k_{i})}{\Delta \omega^{2}+\omega_{1}^{2}}),$$

and further:

(10) $$\Gamma_{i}^{2}/4=\frac{R_{2i}+k_{i}}{k_{i}}\omega_{1}^{2}+{\left(R_{2i}+k_{i}\right)}^{2}.$$

Here, $\delta \omega_{i}$ are the chemical shifts, $f_{i}$ are the proton fractions, $k_{i}$ are the exchange rates, and $R_{2i}$ are the transversal relaxation rates of the individual pools. This solution holds under the assumption of asymmetric populations (i.e., $f_{i}<<1$), and negligible longitudinal relaxation rates $R_{1i}$ of the pools.

This model shows high accuracy compared to numerical solutions of the BM equations. However, the total number of parameters to describe a multi‐pool system is only reduced by the longitudinal relaxation rates of the exchanging pools $R_{1i}$. To reduce the model complexity further, we introduce the following transformation using partial fraction decomposition, which uncouples on‐ and off‐resonant contributions that are entangled as a product of two peaks in Equation (9) (originally referred to as a‐peak and b‐peak [10]):

(11) $$\begin{array}{ll} & R_{\text{ex},i}(\Delta \omega )=R_{\text{ex},i}^{\text{on}}(\Delta \omega )+R_{\text{ex},i}^{\text{off}}(\Delta \omega )\\ & \;=\frac{a_{i}(\omega_{1})\cdot \Delta \omega +b_{i}(\omega_{1})}{\Delta \omega^{2}+\omega_{1}^{2}}+\frac{c_{i}(\omega_{1})\cdot \Delta \omega +d_{i}(\omega_{1})}{\Gamma_{i}^{2}/4+{\left(\Delta \omega -\delta \omega_{i}\right)}^{2}}. \end{array}$$

Solutions are given by:

(12) $$\begin{array}{ll} a_{i}(\omega_{1}) & =\frac{2\cdot \delta \omega_{i}\cdot f_{i}\cdot \omega_{1}^{2}\cdot k_{i}\cdot (\delta \omega_{i}^{2}+R_{2i}(R_{2i}+k_{i}))}{4\omega_{1}^{2}\delta \omega_{i}^{2}+{\left(\delta \omega_{i}^{2}-\omega_{1}^{2}+\Gamma_{i}^{2}/4\right)}^{2}},\\ b_{i}(\omega_{1}) & =\left(\frac{\delta \omega_{i}^{2}-\omega_{1}^{2}+\Gamma_{i}^{2}/4}{2\cdot \delta \omega_{i}}\right)a_{i}(\omega_{1}),\\ c_{i}(\omega_{1}) & =-a_{i}(\omega_{1}),\\ d_{i}(\omega_{1}) & =f_{i}\cdot R_{2i}\cdot \omega_{1}^{2}+\left(2\delta \omega_{i}-\frac{\delta \omega_{i}^{2}-\omega_{1}^{2}+\Gamma_{i}^{2}/4}{2\cdot \delta \omega_{i}}\right)a_{i}(\omega_{1}). \end{array}$$

Defining

(13) $$\begin{array}{ll} A(\omega_{1}) & =\frac{1}{\omega_{1}^{2}}\underset{i}{\sum }a_{i}(\omega_{1}), \end{array}$$

(14) $$\begin{array}{ll} B(\omega_{1}) & =R_{2w}+\frac{1}{\omega_{1}^{2}}\underset{i}{\sum }b_{i}(\omega_{1}) \end{array}$$

one can rewrite

(15) $$R_{1\rho }(\Delta \omega )=\frac{R_{1w}\cdot \Delta \omega^{2}+(A(\omega_{1})\cdot \Delta \omega +B(\omega_{1}))\cdot \omega_{1}^{2}}{\Delta \omega^{2}+\omega_{1}^{2}}+\sum_{i}R_{\text{ex},i}^{\text{off}}(\Delta \omega ).$$

While $A(\omega_{1})$ induces an asymmetry effect around the water resonance, $B(\omega_{1})$ controls the width of the water peak and is directly linked to $T_{2}$‐shortening effects of exchange processes. Note that $B(0)=R_{2,\text{obs}}$ reproduces the Swift–Connick relation [18].

### 2.2 The AB‐MT+n Model

Zaiss et al. [11] also demonstrated that an analytical solution for a MT effect can be found analogously. It requires the following modifications to previous formulas:

(16) $$R_{1,\text{res}}(\Delta \omega )\approx R_{1,\text{obs}}\cdot \cos\theta ,$$

with

(17) $$R_{1,\text{obs}}=\frac{R_{1,w}+f_{\text{MT}}\cdot R_{1,\text{MT}}}{1+f_{\text{MT}}},$$

and

(18) $$R_{1\rho }(\Delta \omega )=R_{\text{eff}}(\Delta \omega )+R_{\text{ex}}^{\text{MT}}(\Delta \omega )+\frac{1}{1+f_{\text{MT}}}\sum_{i}R_{\text{ex},i}(\Delta \omega ).$$

When assuming that close to the water resonance the MT contribution is dominated by the DS effects, that is, one only has to consider large saturation offsets $\Delta \omega >>\omega_{1},R_{1,w},R_{2,w}$, and $k_{\text{MT}}>>r_{1,\text{MT}}$, the MT exchange term can be approximated by:

(19) $$R_{\text{ex}}^{\text{MT}}(\Delta \omega )=\frac{f_{\text{MT}}}{1+f_{\text{MT}}}\cdot r_{1,\text{MT}}(\Delta \omega ),$$

where

(20) $$r_{1,\text{MT}}(\Delta \omega )=R_{1,\text{MT}}-R_{1,w}+R_{\mathrm{rf},\mathrm{MT}}(\Delta \omega ),$$

and $R_{\mathrm{rf},\mathrm{MT}}(\Delta \omega )$ is a suitable absorption line‐shape function (Lorentzian, Gaussian or Super‐Lorentzian).

Equation (19) does not fulfill two properties of the original solution proposed by Zaiss et al. [11], $R_{\text{ex}}^{\text{MT}}(\Delta \omega =0)=0$ and $\partial_{\Delta \omega }\;R_{\text{ex}}^{\text{MT}}(\Delta \omega =0)=0$. These properties can be restored by multiplying Equation (19) with $\Delta \omega^{2}/(\Delta \omega^{2}+\omega_{1}^{2})$, while not affecting the far off‐resonant case. A visual comparison of the described approximation with the original analytical solution for $R_{\text{ex}}^{\text{MT}}$ can be found in Supporting Information Figure S1.

This leads to the final expression for $R_{1\rho }(\Delta \omega )$:

(21) $$\begin{array}{ll} R_{1\rho }(\Delta \omega ) & =\frac{\left(R_{1,\text{obs}}+\frac{f_{\text{MT}}}{1+f_{\text{MT}}}\cdot R_{\text{rf}}^{\text{MT}}(\Delta \omega )\right)\cdot \Delta \omega^{2}}{\Delta \omega^{2}+\omega_{1}^{2}}\\ & \;+\frac{(A(\omega_{1})\cdot \Delta \omega +B(\omega_{1}))\cdot \omega_{1}^{2}}{\Delta \omega^{2}+\omega_{1}^{2}}\\ & \;+\frac{1}{1+f_{\text{MT}}}\underset{i}{\sum }R_{\text{ex},i}^{\text{off}}(\Delta \omega ), \end{array}$$

with

(22) $$\begin{array}{ll} A(\omega_{1}) & =\frac{1}{\omega_{1}^{2}}\underset{i}{\sum }\frac{a_{i}(\omega_{1})}{1+f_{\text{MT}}}, \end{array}$$

(23) $$\begin{array}{ll} B(\omega_{1}) & =R_{2w}+\frac{1}{\omega_{1}^{2}}\underset{i}{\sum }\frac{b_{i}(\omega_{1})}{1+f_{\text{MT}}}. \end{array}$$

We name this model AB‐MT+n, as it includes on‐resonant asymmetry (A) and line‐width (B) effects, MT effects, as well as the off‐resonant contribution of n additional pools.

### 2.3 Compensation of Read‐Out Imperfections

The dynamic model in Equation (4) does not capture two effects previously described by Sun [19] for metric‐based analysis of non‐steady‐state Z‐spectra. They can also be considered for model‐based analysis:

1. Longitudinal relaxation between saturation and read‐out due to delays $t_{\text{delay}}$ arising from required spoilers, field‐of‐view saturation or long read‐outs, reduces the labeling efficiency by a factor $C=\exp(-R_{1,\text{obs}}\cdot t_{\text{delay}})$.
2. The initial magnetization $Z_{i}$ varies due to residual longitudinal magnetization $Z_{\text{res}}(\Delta \omega )=Z(\Delta \omega )\cdot \sqrt{1/\sin^{2}\alpha_{\text{eff}}-1}$ after excitation in gradient‐echo snapshot sequences for an effective flip‐angle $\alpha_{\text{eff}}$.

In Z‐spectroscopy, the signal for a finite sequence of frequency offsets $\Delta \omega_{i}$ is measured. After signal read‐out at one offset, the z‐magnetization will recover by

(24) $$Z_{\text{rec}}(\Delta \omega_{i+1})=1+(Z_{\text{res}}(\Delta \omega_{i})-1)\cdot \exp(-R_{1,\text{obs}}\cdot t_{\text{rec}})$$

until the next saturation phase. That is, the dynamic model has to be solved iteratively. However, for fitting, the model only needs to be calculated at the measured offsets. Then, one can simply use the measured Z value at offset $\Delta \omega_{i-1}$ to calculate $Z_{\text{rec}}(\Delta \omega_{i})$. In total, the dynamic model for acquired Z‐spectra is given by:

(25) $$\begin{array}{cc} Z(\Delta \omega ,t_{\text{sat}}) & =1-C+C\cdot \left(\left(P_{z,\text{eff}}P_{z}Z_{\text{rec}}(\Delta \omega )-Z_{ss}(\Delta \omega )\right)\right.\\ & \;\cdot \left.e^{-R_{1\rho }(\Delta \omega )\;t_{\text{sat}}}+Z_{ss}(\Delta \omega )\right). \end{array}$$

### 2.4 Distinguishability of Exchanging Pools

The previously introduced transformation leading to Equation (21) did not reduce the number of parameters for the CE pools. However, it showed that on‐resonant contributions do not allow for a separation of individual pools along the spectroscopic dimension, as $A(\omega_{1}),B(\omega_{1})$ depend solely on $\omega_{1}$. For single‐$B_{1}$ experiments, they can be treated as constants. If two pools should be distinguishable along the spectroscopic dimension, this has to arise from the off‐resonant contributions.

Consider two fast‐exchanging pools with negligible $R_{2i}$, which fulfill the following conditions:

(26) $$k_{1}\approx k_{2}\approx k_{\text{eff}}\;\text{and}\;\delta \omega_{1}\approx \delta \omega_{2}\approx \delta \omega_{\text{eff}}.$$

Then, they can be approximately described by one effective pool with exchange rate $k_{\text{eff}}$ and chemical shift $\delta \omega_{\text{eff}}$ and their proton fractions are additive $f_{\text{eff}}=f_{1}+f_{2}$. This fusion of pools reduces the number of distinguishable pools n in the sum of off‐resonant contributions. It is often implicitly assumed to group different metabolites by their predominantly exchanging functional group (e.g., hydroxyl, amine,…).

In addition, for very fast exchanging pools, the off‐resonant contribution $R_{\text{ex},i}^{\text{off}}$ is suppressed by $k_{i}^{2}$ compared to the on‐resonant contribution $R_{\text{ex},i}^{\text{on}}$, and might be completely omitted. The AB‐MT part therefore represents a basis set of five parameters $\left\{A,B,f_{\text{MT}},R_{2,\text{MT}},\delta \omega_{\text{MT}}\right\}$ required for all Z‐spectra, which can be extended by $R_{\text{ex},i}^{\text{off}}$ terms of n distinct observable pools, each adding three to four more parameters $\left\{\delta \omega_{i},f_{i},k_{i},(R_{2,i})\right\}$ to the total model. A detailed discussion on appropriately choosing the number of effectively distinguishable pools n will follow in subsequent paragraphs.

## 3 Methods

### 3.1 Simulation of Multi‐Pool Z‐Spectra

Steady‐state Z‐spectra were simulated using Pulseq‐CEST [20] and fitted with multiple analytical models to reason appropriate model selection for quantitative Z‐spectroscopy. Rician noise was added to mimic realistic conditions and test robustness of the fitting. Given a noise‐free Z‐spectrum with signal intensities, the noisy signal can be modeled by drawing samples from the Rice distribution. The probability density function (pdf) of the Rice distribution is given by [21]

(27) $$f(x;Z,\sigma )=\frac{x}{\sigma^{2}}\exp\left(-\frac{x^{2}+Z^{2}}{2\sigma^{2}}\right)I_{0}\left(\frac{xZ}{\sigma^{2}}\right),$$

where $I_{0}$ is the modified Bessel function of the first kind and σ represents the noise standard deviation. We employed a gradient‐based L‐BFGS‐B solver from SciPy [22, 23] to fit Z‐spectra with the analytical steady‐state solution (Equation (5)). Different models for $R_{1\rho }$ were inserted in this equation, including the full analytical model proposed by Zaiss et al. [10, 11] and the here proposed AB‐MT+n model with different numbers of explicit pools considered. Fit parameters were initialized with corresponding simulation values. Bounds were set to ±50% deviation from initial value.

First, a four‐pool toy model was simulated, comprising one water‐pool and one MT‐pool, as well as two CE pools which share the same variable exchange rate $k=k_{1}=k_{2}$. Further, a Lorentzian MT line‐shape was assumed, and $B_{0}=9.4\;T$ and $B_{1}=1.0\;\mu$T. Exchange rates varied from 200 to 20,000 Hz. Hundred different noise patterns with σ=0.01 were generated and added to the simulated spectra.

Second, a GM multi‐pool model as proposed by van Zijl et al. [24] was simulated at $B_{0}=3$ T and multiple saturation amplitudes ($B_{1}=0.5,1.0,2.0,4.0\;\mu$T). It consists of a water pool, one MT pool with Super‐Lorentzian line‐shape, one combined relayed nuclear overhauser effect (rNOE) pool, as well as amide, amine, guanidino, hydroxyl pools. To account for the noise‐dependence of model‐order selection, 100 noisy Z‐spectra (σ=0.005) were generated for each $B_{1}$‐value and fitted with the analytical AB‐MT+n model, while varying the number of explicitly modeled CEST pools (n). Pools were added in order of decreasing exchange‐time scale (fastest pools removed first when reducing n). For every fitted spectrum, BIC was calculated, and the BIC‐optimal model order was recorded. Constant simulation parameters of both models can be found in Supporting Information Table S1.

To assess how well the n fitted explicit components represented the true off‐resonant exchange contributions, we additionally evaluated the isolated ground‐truth off‐resonant $R_{\text{ex},i}^{\text{off}}$ terms of the simulated GM pools from Equations (11) and (12),

(28) $$R_{\text{ex},i}^{\text{off}}(\Delta \omega )=\frac{c_{i}(\omega_{1})\Delta \omega +d_{i}(\omega_{1})}{\Gamma_{i}^{2}/4+{\left(\Delta \omega -\delta \omega_{i}\right)}^{2}}.$$

We then compared these to the effectively fitted off‐resonant contributions ${\hat{R}}_{\text{ex},j}^{\text{off}}$ (j=1,…,n). For each fit, normalized root‐mean‐square error (NRMSE) between fitted ${\hat{R}}_{\text{ex},j}^{\text{off}}$ and corresponding ground‐truth $R_{\text{ex},i}^{\text{off}}$ was calculated to quantify component recovery:

(29) $$\text{NRMSE}=\frac{\sqrt{\frac{1}{N}\sum_{k=1}^{N}{\left({\hat{R}}_{\text{ex},j}^{\text{off}}(\Delta \omega_{k})-R_{\text{ex},i}^{\text{off}}(\Delta \omega_{k})\right)}^{2}}}{\underset{k}{\max}\left|R_{\text{ex},i}^{\text{off}}(\Delta \omega_{k})\right|}.$$

### 3.2 Mixed Glc/Glu Phantoms

All experiments were performed on a BioSpec Maxwell 9.4 T small animal MR system (Bruker BioSpin GmbH, Ettlingen, Germany) with ParaVision 360 3.6, equipped with an actively shielded gradient system (maximum gradient amplitude of 961 mT/m, slew rate = 4600 T/m/s), and a dual setup of a 82 mm transmit‐only volume resonator and a four‐element receive‐only cryogenic probe.

In total, nine different phantoms were prepared: D(+)‐Glucose (Glc, VWR International) and L‐Glutamic acid (Glu, Sigma Life Science) were dissolved at three different concentrations each (10, 20, 40 mM) in phosphate buffered saline (PBS) with 1% agarose (Carl Roth) added. Room temperature ($20^{\circ }C$) was maintained for all measurements. Each phantom was measured with two different saturation schemes: (a) cw‐CEST saturation with $t_{\text{sat}}=4000$ ms and $B_{1}=2\;\mu$T, $T_{R}=5000\;\text{ms}$, (b) cw‐CESL saturation with $t_{\text{sat}}=500$ ms and $B_{1}=5\;\mu$T, $T_{R}=2000$ ms. 191 frequency offsets were measured: −50 ppm, −49 ppm, …, −5 ppm, −4.9 ppm, …, 4.9 ppm, 5 ppm, …, 49 ppm, 50 ppm. Spectra were normalized with a reference scan without saturation and $T_{R}=20$ s. All other imaging parameters were unaltered: Gradient‐echo snapshot sequence, 40×40 image matrix, 0.5×0.5 mm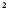 resolution, 2 mm slice thickness, $T_{E}=2.9$ ms, effective $T_{E}=84.7$ ms.

To correct for $B_{0}$‐ and $B_{1}^{\left(+\right)}$‐inhomogeneities, we performed a WASABI [25] experiment, and corrected Δω and $\omega_{1}$ pixel‐wise. $R_{1,\text{obs}}$ was provided through an additionally acquired $T_{1}$‐map (inversion recovery, 20 geometrically‐spaced inversion times up to 8000 ms).

The obtained spectra were fitted pixel‐wise with the full analytical model [10, 11] (Equations ((4), (5), (6), (7), (8), (9), (10))), the AB‐MT+n model (Equations (25) and (21)), as well as a Lorentzian model [24]:

(30) $$Z(\Delta \omega )=Z_{\text{base}}-\underset{i}{\sum }\frac{a_{i}\cdot \Gamma_{i}^{2}/4}{\Gamma_{i}^{2}/4+{\left(\Delta \omega -\delta \omega_{i}\right)}^{2}},$$

with the peak height $a_{i}$, width $\Gamma_{i}$, center $\delta \omega_{i}$, and $0<Z_{\text{base}}<1$ accounting for incomplete recovery of Z‐magnetization. All fitted models consider either three (AB‐MT+1: water, MT, Glu) or four distinct pools (AB‐MT+2, four‐pool Lorentzian: water, MT, Glc, Glu), with one effective glucose pool, combining all four glucose resonances [26]. For the AB‐MT+n model, a Gaussian line‐shape for the MT contribution was assumed. Additionally, we calculated area under the curve (AUC) of MTR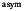 between 0.5 and 2.0 ppm for Glc and between 2.5 and 3.5 ppm for Glu [27]. We performed a linear regression between fitted model parameters/AUC values (observables) and metabolite concentrations (dependencies) and quantified linearity using the coefficient of determination ($R^{2}$). To improve algorithmic stability, we fitted each spectrum with ten random initial parameter values within reasonable bounds and chose the lowest‐residue solution as the final result. Used boundary values for each model can be found in Supporting Information Table S2.

### 3.3 GlucoCESL in Mouse Brain

Animal experiments were carried out according to local animal welfare guidelines and were approved by local authorities (ID: 81‐02.04.2021.A042). One healthy mouse was measured (male C57BL/6J, housed under a 12 h light‐dark cycle, provided with food and water ad libitum). Z‐spectra were acquired pre and post administration of an IV bolus of 6 μmol/g Glc. CESL saturation was applied: $B_{1}=5\;\mu$T, $t_{\text{sat}}=500$ ms, 41 equally spaced frequency offsets (−6–+6 ppm). One coronal slice covering the whole brain was imaged using a segmented FLASH sequence: 100×100 image matrix, 0.2×0.2 mm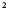 resolution, 1 mm slice thickness, $T_{E}=2.8$ ms, 2 segments of 40 k‐space lines, homodyne k‐space completion, $T_{R}=3$ s. Spectra were normalized with a reference scan without saturation and $T_{R}=10$ s, four averages. $B_{0}$‐ and $B_{1}$‐maps (WASABI [25]) and $T_{1}$‐maps (inversion recovery, 12 geometrically‐spaced inversion times up to 6000 ms) were acquired before bolus administration. The AB‐MT model (Equations (25) and (21), with no explicit pool n=0 considered) was fitted pixel‐wise.

## 4 Results

### 4.1 Validation of the AB‐MT+n Model on Simulated Data

**FIGURE 1:**
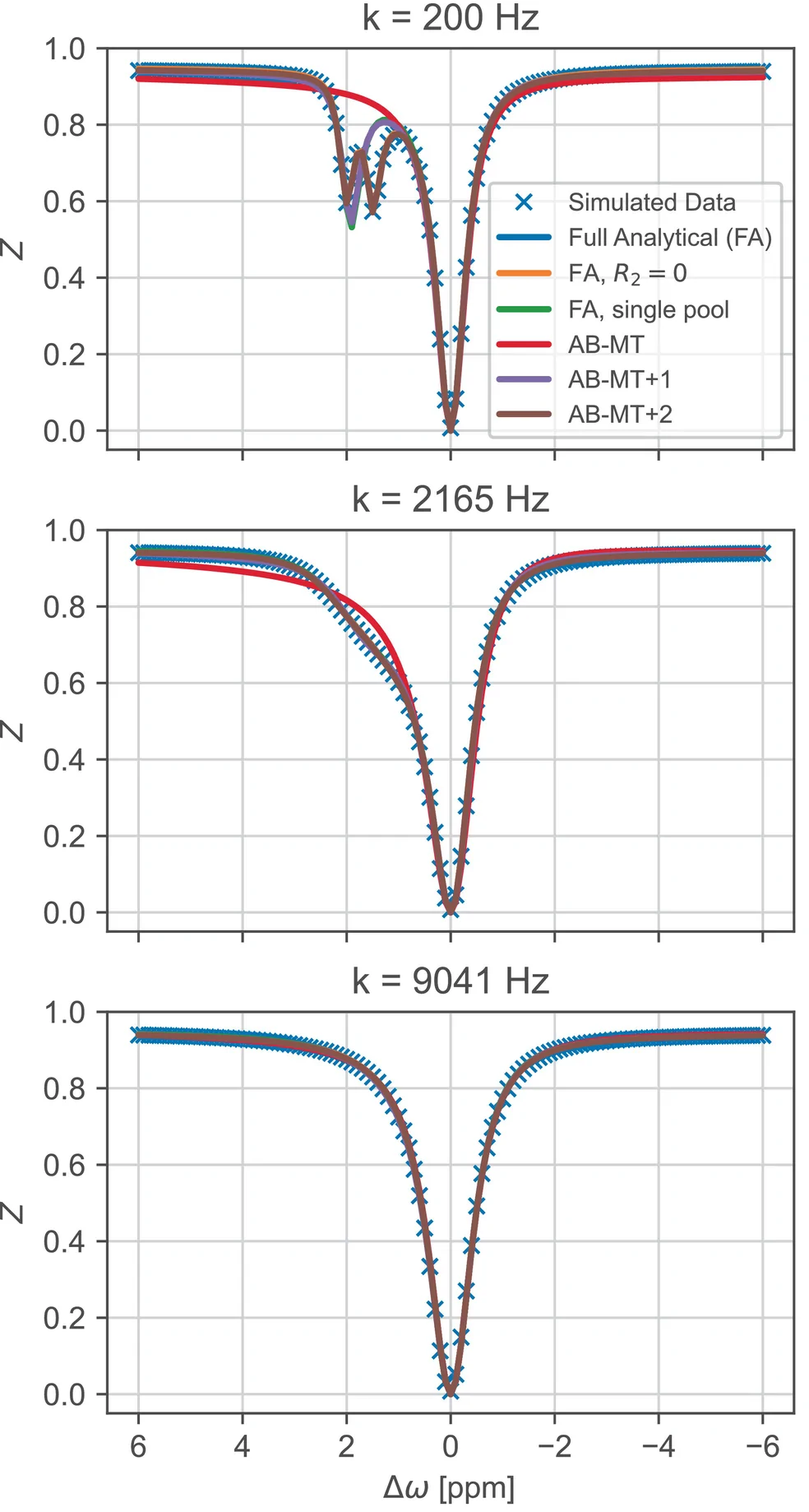
Mean of the 100 generated noisy spectra and comparison of fitted models for k=200, k=2165, k=9041 Hz (top to bottom).

In a first step, we fitted the AB‐MT+n model to simulated spectra obtained by numerically solving the BM equations for a four‐pool toy model along with the full analytical model from Zaiss et al. [10, 11] to validate its accuracy. Mean spectra and fit results of the 100 generated noisy spectra for CE rates of k=200, k=2165, k=9041 Hz are shown in Figure 1. In any case, the AB‐MT+2 proved equivalently accurate to the full analytical model, with and without consideration of $R_{2}$. The $R_{2i}$‐dependency has no distinct effect along the spectroscopic axis (Equation (12)), and thus cannot be resolved in single‐$B_{1}$ experiments. It was therefore omitted for the AB‐MT+n model as well. The additional approximation of the MT effect that distinguishes the AB‐MT+2 model from the full analytical model, did not harm the accuracy of the model, but reduced the degrees of freedom further.

For the lowest exchange rate, an explicit consideration of two pools in the analytical models was required to accurately model the simulated data. In the middle case, both peaks melted together and could be modeled by one effective pool. With further growing exchange rate, the AB‐MT model without any explicit pool consideration became equivalent to all other models.

**FIGURE 2:**
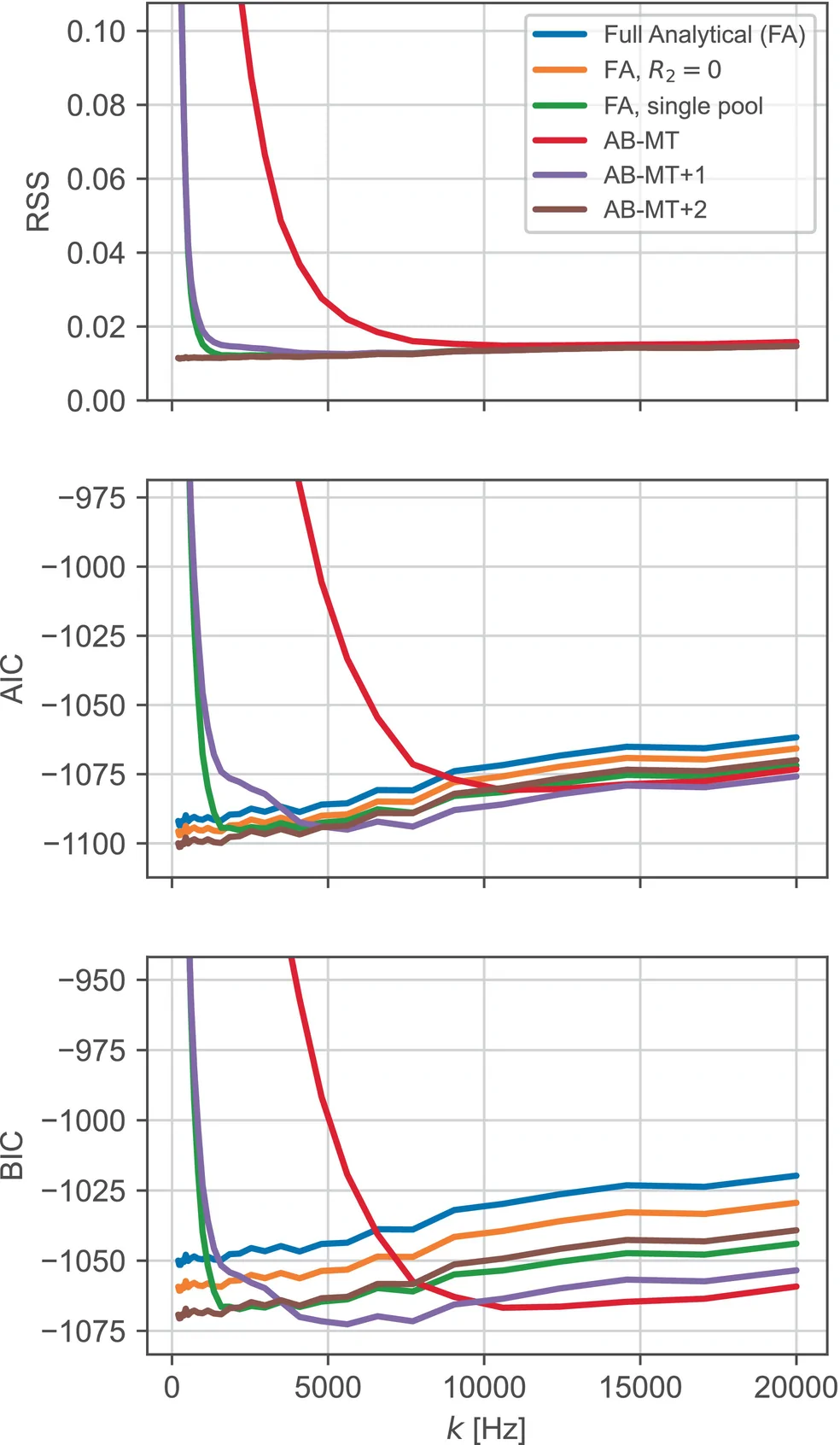
Mean residual sum of squares (RSS), Akaike information criterion (AIC) and Bayesian information criterion (BIC) of the 100 generated noisy spectra fitted with different models in dependence of the common exchange rate k.

To investigate the growing equivalence of the different models in more detail, Figure 2 depicts residual sums of squares (RSS), Akaike information criterion (AIC), and Bayesian information criterion (BIC) in dependency of the common exchange rate of the two exchanging pools. With growing exchange rate, the RSS of all fitted models aligned. Consequently, AIC and BIC showed a growing preference towards simpler models for inverse problem formulation [28]. CE pools became increasingly indistinguishable from each other and even the water pool itself, that is, the amount of restorable information decreased with increasing exchange rate.

**FIGURE 3:**
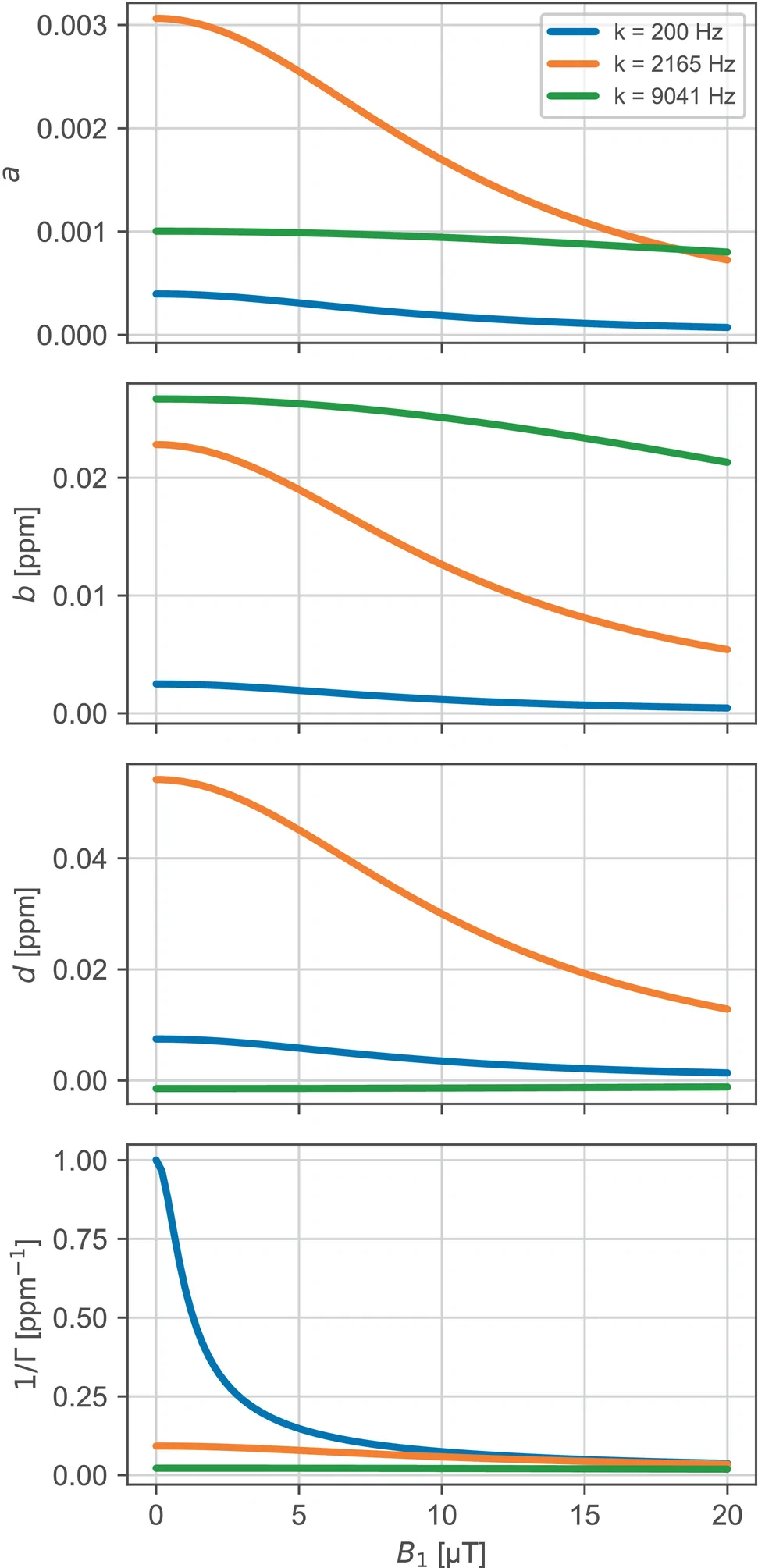
Functions $a(B_{1}),b(B_{1}),d(B_{1}),\Gamma (B_{1})$ from Equations (10) and (12) for exchanging pool (b) from Table S2 and k=200, k=2165, k=9041 Hz.

If pools cannot be separated along the spectroscopic axis, it is a commonly chosen strategy to perform Z‐spectroscopy at multiple saturation strengths (QUESP [29, 30]). This effectively represents a sampling of the functions $a(B_{1}),b(B_{1}),d(B_{1}),\Gamma (B_{1})$ from Equations (10) and (12) which describe all $B_{1}$‐dependency of the model. These functions must vary to a sufficient amount over the course of a realistically measurable $B_{1}$‐range. Figure 3 shows that in the slow exchange regime (k=200 Hz) $\Gamma (B_{1})$, and in the intermediate to fast exchange regime (k=2165 Hz) $a(B_{1}),b(B_{1}),d(B_{1})$ varied the most. In either case, this allows for quantitative measurement of proton fractions, exchange and relaxation rates. On the other hand, there is no further benefit from QUESP acquisition schemes for very fast exchanging pools (k=9041 Hz), as all $B_{1}$‐dependent functions behaved almost constant over a wide range of $B_{1}$‐values.

### 4.2 Quantifying Mixed Glc/Glu Phantoms With the AB‐MT+n Model

**FIGURE 4:**
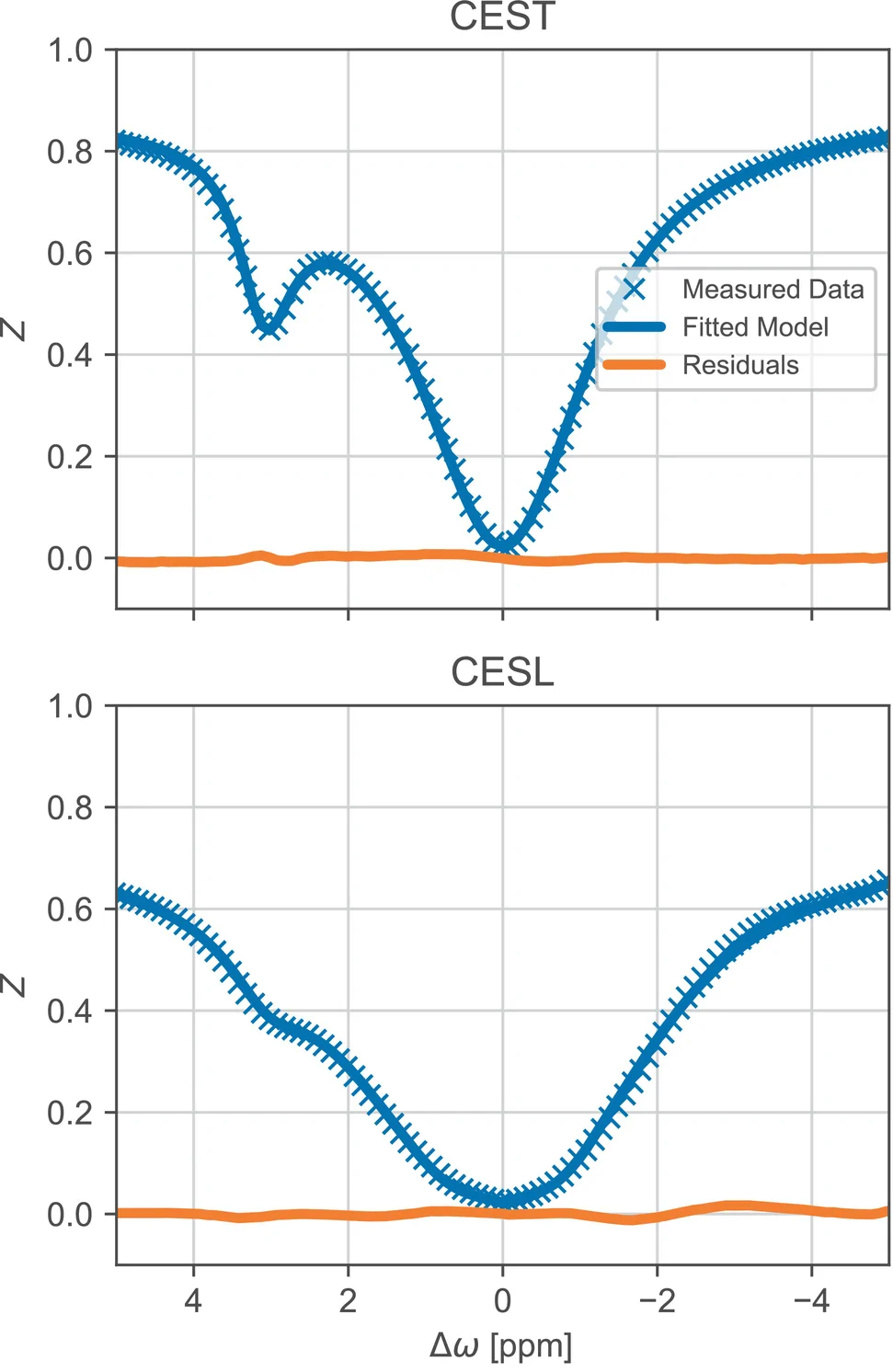
Mean Z‐spectra, fits and offset‐wise residuals of the AB‐MT+2 model for mixed Glc/Glu phantoms (40 mM each) with cw‐CEST saturation with $t_{\text{sat}}=4000$ ms and $B_{1}=2\;\mu$T (top) and cw‐CESL saturation with $t_{\text{sat}}=500$ ms and $B_{1}=5\;\mu$T (bottom).

Next, we investigated how meaningful contrasts can be restored from measured Z‐spectra using the AB‐MT+n model. Therefore, acquired Z‐spectra of mixed Glc/Glu phantoms were fitted with different models, including an AB‐MT+1, an AB‐MT+2, the full analytical model [10, 11] and a four‐pool Lorentzian model. Figure 4 depicts two average fits of the AB‐MT+2 model to Z‐spectra of phantoms with 40 mM of Glc and Glu, acquired after CEST saturation ($t_{\text{sat}}=4000$, $B_{1}=2\;\mu$T), and CESL saturation ($t_{\text{sat}}=500$ ms, $B_{1}=5\;\mu$T). Negligible deviations indicated a sufficient accuracy of the AB‐MT model with two explicit pools, combining all four glucose resonances in one effective pool. This held for both saturation schemes.

**FIGURE 5:**
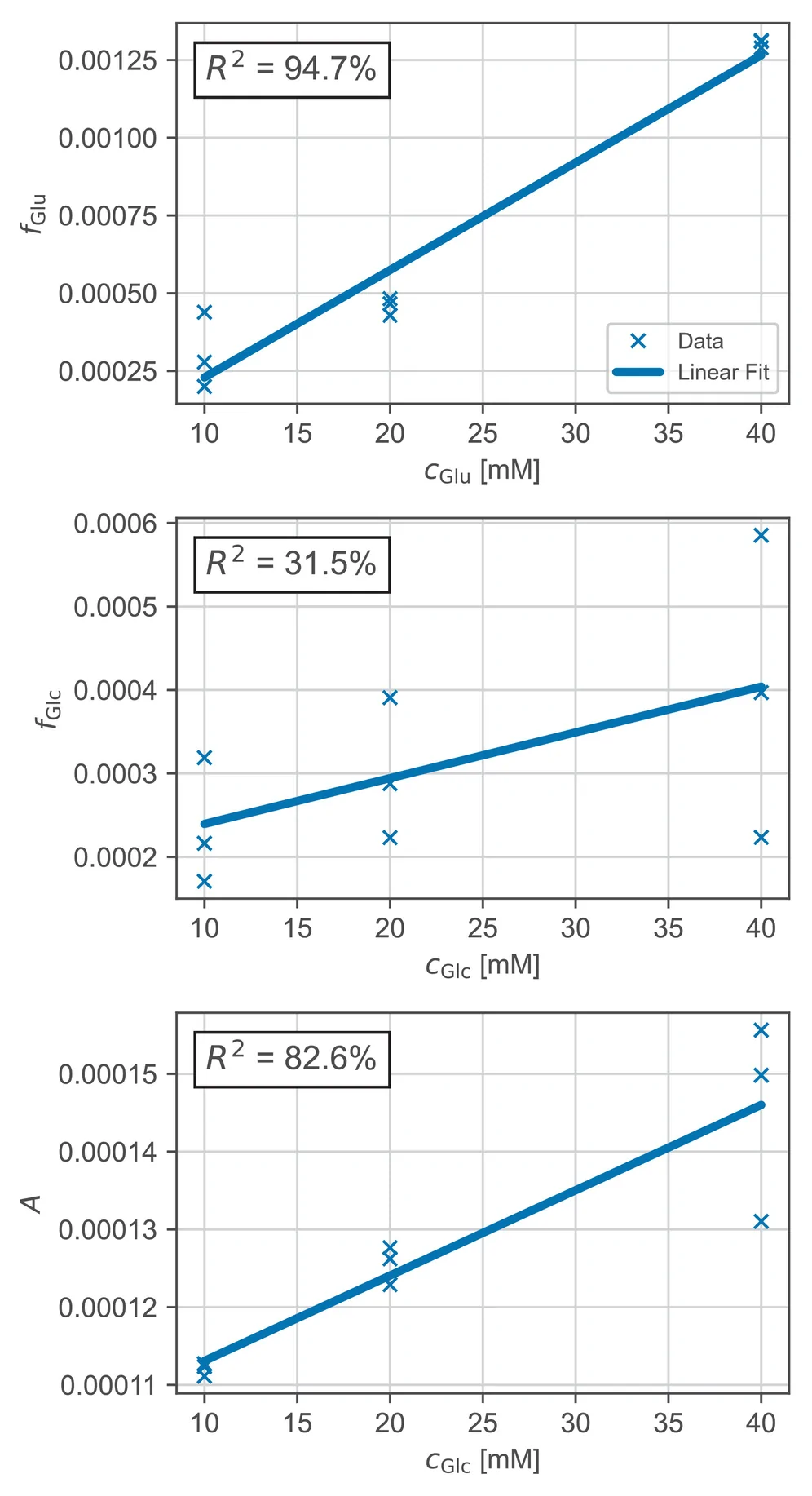
Fitted proton fractions $f_{\text{Glu}}$ and $f_{\text{Glc}}$ in dependency of the respective concentrations, as well as the asymmetry parameter A in dependency of the Glc concentration. The three data points per concentration represent the different concentrations of the other metabolite. Common linear regression curves and coefficients of determination ($R^{2}$) are displayed.

Besides model accuracy, robustness to confounding factors like interference of multiple metabolites is key to ensure reproducibility of results. Therefore, fit results were more thoroughly evaluated by plotting obtained proton fractions in dependency of the corresponding metabolite concentrations, along with linear regression curves and coefficients of determination ($R^{2}$) which indicate how well the proton fractions actually probe the concentrations. Figure 5 illustrates this for the CESL preparation scheme.

While $f_{\text{Glu}}$ was highly linear to the underlying Glu concentration, $f_{\text{Glc}}$ poorly scaled with the Glc concentration. On the other hand, the asymmetry parameter of the on‐resonant contribution A showed much higher linearity to the Glc concentration.

**FIGURE 6:**
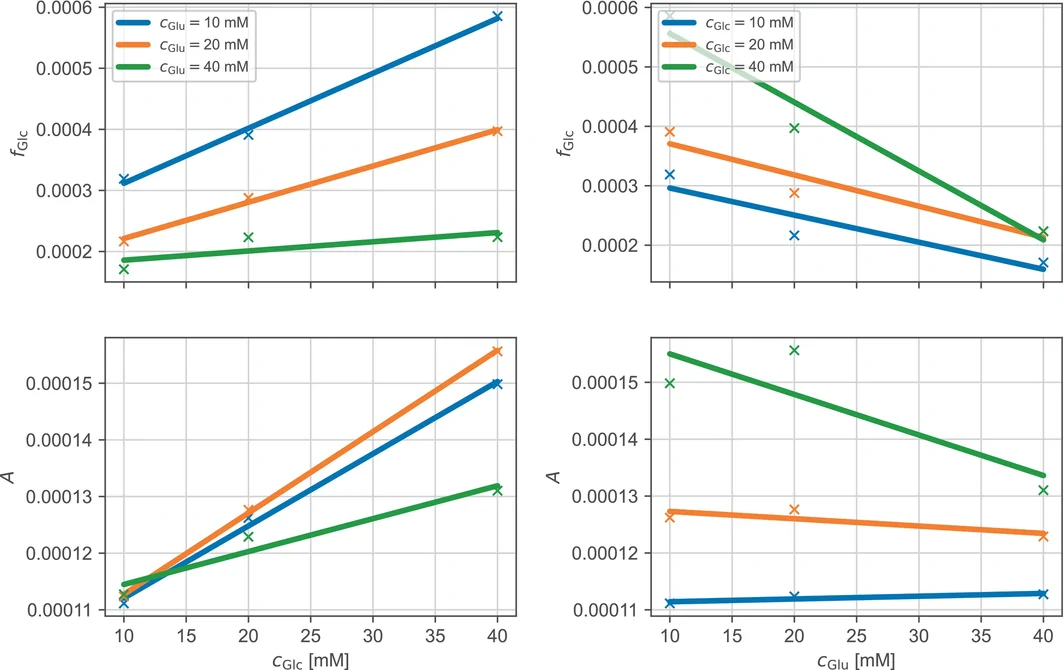
Fitted proton fraction $f_{\text{Glc}}$ and asymmetry parameter A in dependency of the Glc concentration (left) and Glu concentration (right). Linear regression was performed separately for the different metabolite concentrations, marked with different colors of the data points and regression curves.

To investigate what effect causes the unexplained variance of $f_{\text{Glc}}$ and A in Figure 5, linear regression on these was also performed for all Glu concentrations separately, displayed in Figure 6. The variance of the data points around the regression curves was visibly reduced, hinting that most of the previous variance apparent for Glc parameters arises from interference with Glu parameters. Figure 6 also shows the dependency of $f_{\text{Glc}}$ and A on the Glu concentration. Higher glutamate concentration impacted both glucose‐related readouts. Per 1mM glutamate increase, $f_{\text{Glc}}$ reduced by −1.43% ($c_{\text{Glc}}=10$ mM), −1.34% ($c_{\text{Glc}}=20$ mM), and −1.98% ($c_{\text{Glc}}=40$ mM), respectively (mean: $-1.58\pm 0.34\%\;{\text{mM}}^{-1}$). Over the same range, A changed by +0.04%, −0.10%, and −0.48% (mean: $-0.18\pm 0.27\%\;{\text{mM}}^{-1}$). Thus, A shows lower glutamate interference than $f_{\text{Glc}}$, with an approximately 8.9‐fold smaller average relative change per mM glutamate.

$R^{2}$ values were calculated for all fitted models (AB‐MT+1, AB‐MT+2, full analytical, four‐pool Lorentzian) and MTR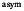 for both saturation schemes. The results for all analysis methods are given in Table 1. Comparing AB‐MT+1 and AB‐MT+2, $f_{\text{Glu}}$ and A showed lower linearity to metabolite concentrations when no explicit glucose pool was considered. On the other hand, B showed slightly higher linearity within the AB‐MT+1 model. The four‐pool Lorentzian and full analytical model showed similar quantification quality for Glu compared to AB‐MT models but considerably worse results for Glc. This was also observed for the metric‐based analysis using MTR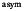, where the quantification of Glc completely broke down for high Glu concentration. For all analysis methods, the quantification quality for Glu was consistently better for lower $B_{1}$ while it improved for Glc with higher $B_{1}$.

**TABLE 1:** Coefficient of determination ($R^{2}$) measuring linearity between all calculated metabolite specific observables and corresponding concentration for the two different saturation schemes.

Observable (dependency)	$t_{\text{sat}}=4000$ ms	$t_{\text{sat}}=500$ ms
	$B_{1}=2\;\mu$T	$B_{1}=5\;\mu$T
**AB‐MT+1**		
$f_{\text{Glu}}(c_{\text{Glu}})$	97.9%	87.5%
$A(c_{\text{Glc}})$	30.2%	63.6%
$B(c_{\text{Glc}})$	64.5%	31.4%
$A(c_{\text{Glc}})$		
For $c_{\text{Glu}}=10$ mM	99.9%	99.97%
For $c_{\text{Glu}}=20$ mM	99.4%	99.4%
For $c_{\text{Glu}}=40$ mM	89.5%	78.0%
**AB‐MT+2**		
$f_{\text{Glu}}(c_{\text{Glu}})$	99.2%	94.7%
$f_{\text{Glc}}(c_{\text{Glc}})$	20.4%	31.5%
$A(c_{\text{Glc}})$	35.2%	82.6%
$B(c_{\text{Glc}})$	44.5%	22.3%
$A(c_{\text{Glc}})$		
For $c_{\text{Glu}}=10$ mM	97.4%	99.6%
For $c_{\text{Glu}}=20$ mM	99.1%	99.95%
For $c_{\text{Glu}}=40$ mM	90.2%	93.7%
$f_{\text{Glc}}(c_{\text{Glc}})$		
For $c_{\text{Glu}}=10$ mM	98.0%	99.5%
For $c_{\text{Glu}}=20$ mM	91.7%	99.5%
For $c_{\text{Glu}}=40$ mM	55.0%	57.6%
**Full analytical**		
$f_{\text{Glu}}(c_{\text{Glu}})$	93.7%	97.6%
$f_{\text{Glc}}(c_{\text{Glc}})$	18.3%	13.2%
$f_{\text{Glc}}(c_{\text{Glc}})$		
For $c_{\text{Glu}}=10$ mM	98.7%	99.0%
For $c_{\text{Glu}}=20$ mM	92.7%	99.2%
For $c_{\text{Glu}}=40$ mM	8.3%	22.6%
**Four‐pool Lorentzian**		
$a_{\text{Glu}}(c_{\text{Glu}})$	99.3%	88.1%
$a_{\text{Glc}}(c_{\text{Glc}})$	25.7%	44.3%
$a_{\text{Glc}}(c_{\text{Glc}})$		
For $c_{\text{Glu}}=10$ mM	99.98%	99.6%
For $c_{\text{Glu}}=20$ mM	99.9%	87.9%
For $c_{\text{Glu}}=40$ mM	15.0%	44.4%
**MTR** 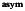		
2.5–3.5 ppm$\;(c_{\text{Glu}})$	97.2%	84.1%
0.5–2.0 ppm$\;(c_{\text{Glc}})$	39.1%	47.3%
0.5–2.0 ppm$\;(c_{\text{Glc}})$		
For $c_{\text{Glu}}=10$ mM	99.5%	99.99%
For $c_{\text{Glu}}=20$ mM	99.7%	96.0%
For $c_{\text{Glu}}=40$ mM	3.2%	6.7%

### 4.3 Applicability of the AB‐MT+n Model on a Realistic GM Model

Previous sections mainly focused on validation of the AB‐MT+n on dedicated exchange systems. In a next step, we aimed to demonstrate the applicability of the model when approaching in vivo conditions. For that purpose, we first investigated the applicability of the AB‐MT+n model to a simulated GM seven‐pool model (water, MT, rNOE, amide, amine, guanidino, hydroxyl). We fitted 100 simulated noisy spectra with the AB‐MT+n model of different orders (n=0,…,5). For each model order, the BIC was calculated, and the BIC‐optimal n was recorded for each individual fit.

**FIGURE 7:**
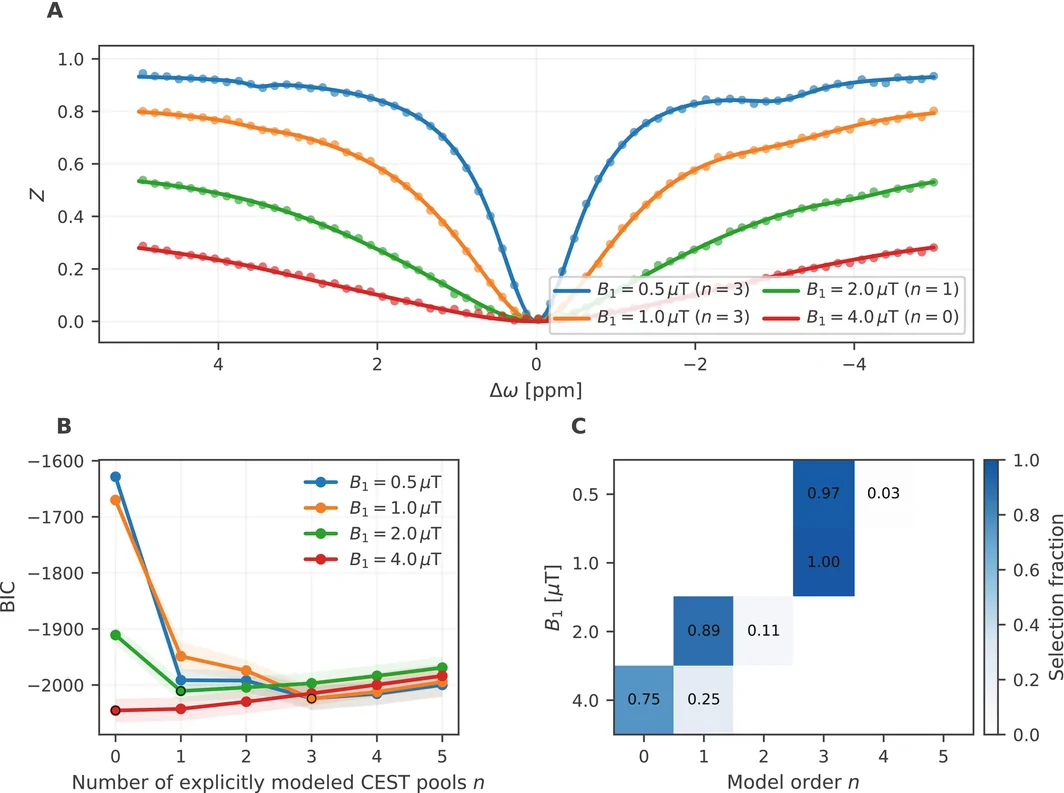
AB‐MT+n model‐order selection for simulated GM [24] spectra. (A) Representative noisy Z‐spectra of the simulated GM model at different saturation powers and fits of selected model order n. (B) Mean BIC across 100 fitted noisy spectra as a function of the number of explicitly modeled off‐resonant pools n. Shaded regions indicate one standard deviation. (C) BIC model‐order selection frequencies. All nonzero selection fractions are annotated.

Figure 7A displays representative noisy spectra and fits for the BIC‐optimal number of pools n. The BIC curves and model‐order selection frequencies are summarized in Figure 7B and C. Consistently across all $B_{1}$, BIC favored reduced models with n<5, indicating that additional explicit pools did not always provide sufficient explanatory benefit to justify their additional degrees of freedom. The BIC‐optimal model order decreased further at higher saturation power: n=3 at $B_{1}=0.5$ and $B_{1}=1.0\;\mu$T, n=1 at $B_{1}=2.0\;\mu$T, and n=0 at $B_{1}=4.0\;\mu$T. The corresponding BIC selection fractions were 0.97, 1.00, 0.89, and 0.75, respectively. Selection frequencies <1.00 indicate that noise can confound model‐order selection, particularly when neighboring model orders yield similar fit quality.

**FIGURE 8:**
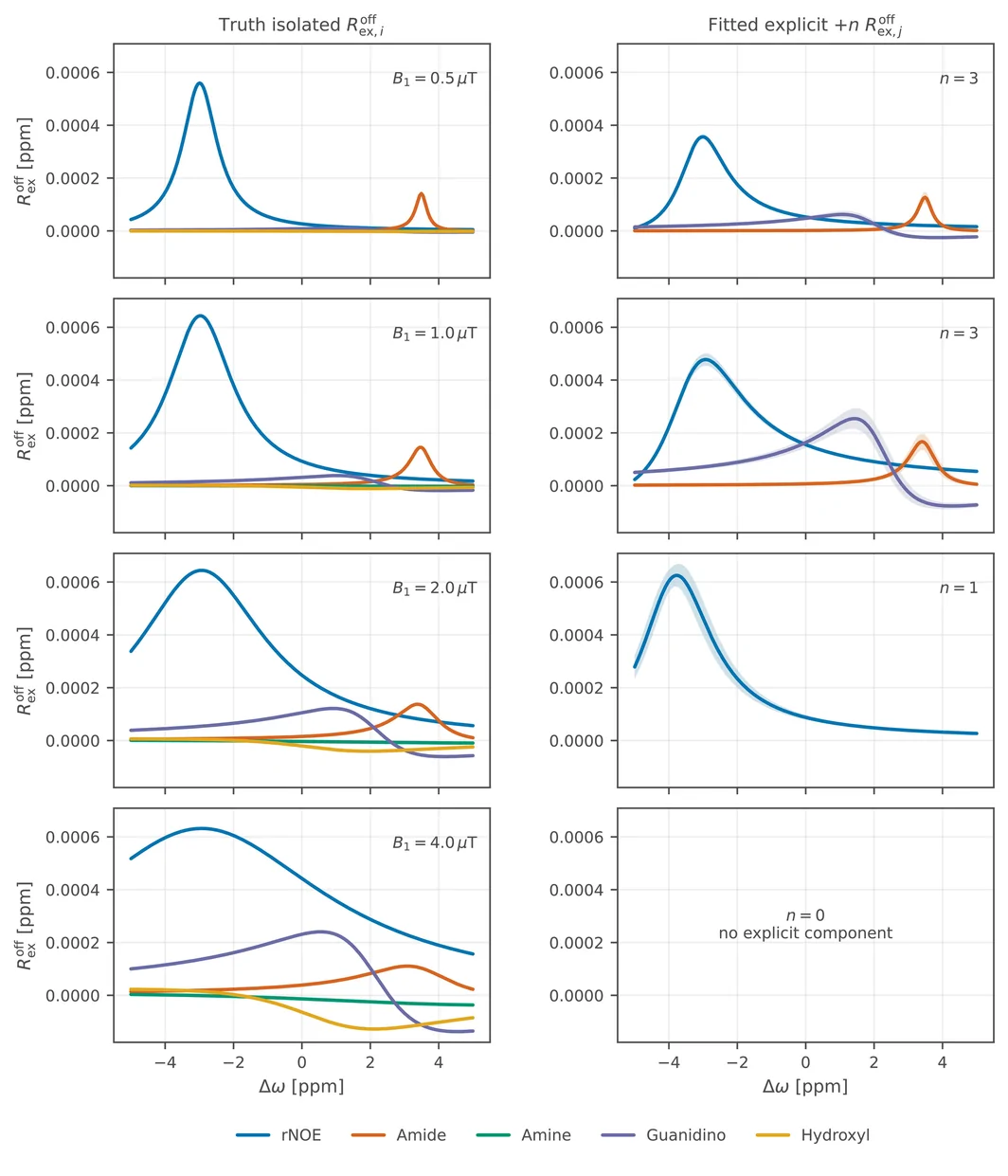
Validation of explicit off‐resonant components in the GM simulation. Left column: Isolated ground‐truth $R_{\text{ex},i}^{\text{off}}$ contributions of the simulated exchange pools. Right column: Fitted n explicit $R_{\text{ex},j}^{\text{off}}$ components for the BIC‐optimal model order at the corresponding $B_{1}$. Solid lines indicate mean of all fits, while shaded regions indicate one standard deviation.

Next, we compared the fitted explicit $R_{\text{ex},j}^{\text{off}}$ components with the noise‐free ground‐truth $R_{\text{ex},i}^{\text{off}}$ contributions of the simulated GM pools (Figure 8). At $B_{1}=0.5\;\mu$T, n=3 explicit components were retained corresponding to rNOE, amide, and guanidino. The rNOE and amide contributions were recovered accurately, with NRMSE values of 0.10±0.01 and 0.04±0.02, respectively. The additional guanidino component showed substantially larger deviations from the isolated ground‐truth contribution (NRMSE 2.68±0.44), indicating that it should not be interpreted as robust quantitative guanidino recovery. At $B_{1}=1.0\;\mu$T, the BIC analysis also indicated three explicit components to be optimal. The rNOE and amide contributions remained well represented, with NRMSE values of 0.13±0.01 and 0.10±0.05, respectively, whereas the guanidino component again showed large deviations (NRMSE 2.76±0.33). At $B_{1}=2.0\;\mu$T, only one explicit component was retained, corresponding to rNOE, whose recovery remained spectrally similar but less accurate (NRMSE 0.24±0.02).

### 4.4 Measuring Glucose Uptake in the Brain Using the AB‐MT Model

**FIGURE 9:**
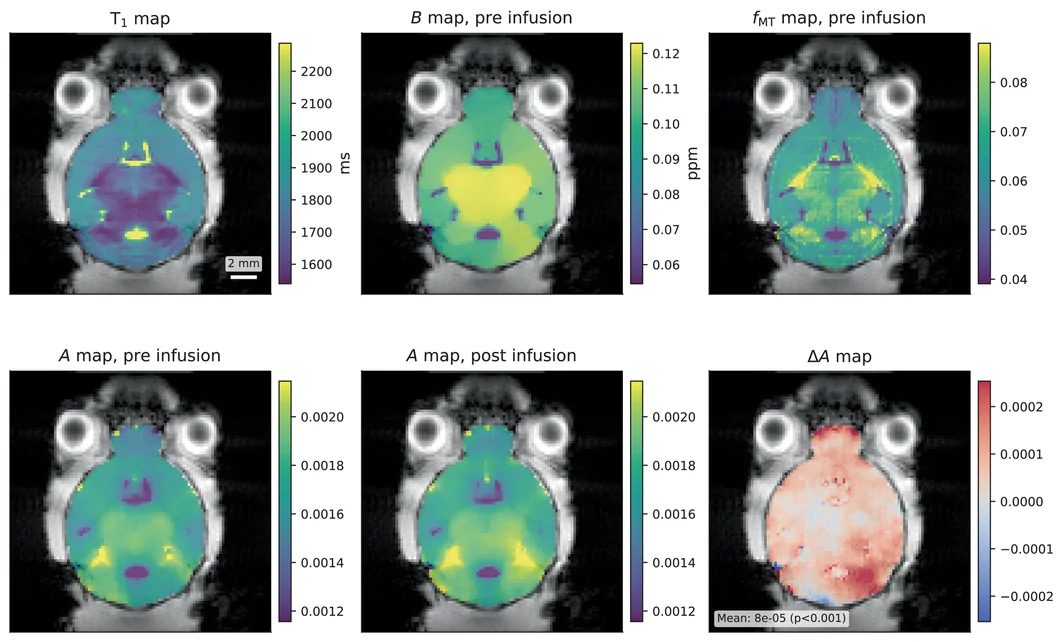
Brain glucoCESL results for a representative mouse. Shown are the fitted $T_{1}$ (inversion recovery model), B, and $f_{\text{MT}}$ (AB‐MT model) parameter maps, together with the fitted A maps before infusion, after infusion, and the corresponding difference map ($\Delta A=A_{\text{post}}-A_{\text{pre}}$). A values were shown to be significantly larger after infusion using a one‐sided *t*‐test (p<0.001).

Finally, we examined the feasibility of the AB‐MT+n model to quantify glucose uptake in a glucoCESL experiment in healthy mice. The chosen saturation scheme ($B_{1}=5\;\mu$T, $t_{\text{sat}}=500$ ms) was previously described [26] as highly sensitive to glucose‐induced changes. Phantom measurements in Section 4.2 indicated a strong linear dependence of the asymmetry parameter A on glucose concentration for this saturation scheme. We therefore focused on the AB‐MT component and quantified glucose uptake through $\Delta A=A_{\text{post}}-A_{\text{pre}}$. As shown in Figure 9, the post‐infusion A map showed higher values across large parts of the brain than the pre‐infusion map. Within the whole‐brain ROI, the mean ΔA was significantly greater than zero in a one‐sided t‐test, indicating detectable glucose‐related contrast in the brain after infusion. Additionally, baseline maps of A, B, and $f_{\text{MT}}$ revealed distinct quantitative tissue contrasts. Remarkably, $T_{1}$ and $f_{\text{MT}}$ exhibited a close inverse spatial relationship, as expected from Equation (17).

## 5 Discussion

### 5.1 The AB‐MT+n Framework and the Information Content of Z‐Spectra

Simulations showed that the AB‐MT+n model essentially allows to extract the full information that is practically accessible from Z‐spectra, while utilizing a minimal set of parameters. In the slow‐exchange regime the AB‐MT+2 variant matched the full analytical model only when two explicit pools were represented. In the intermediate regime, a single effective pool sufficed, and for very fast exchange the AB‐MT form became equivalent to the more extensive models.

This observation matches previously described coalescence effects [24, 31] when approaching the fast‐exchange regime. However, the AB‐MT+n model describes this observation more nuanced, as already pointed out in Section 2.4: Off‐resonant contributions eventually coalesce when exchange rates and chemical shifts become comparable on the exchange rate scale, but the apparent coalescence of exchange effects and DS effects is rather caused by a suppression of off‐resonant effects compared to on‐resonant effects described by the A and B parameters of the AB‐MT+n model. On‐resonant effects are always present, no matter the exchange regime, but become more dominant compared to off‐resonant effects in the fast‐exchange limit.

Evaluation techniques that assume locality of the exchange effects like multi‐pool Lorentzian fits or AUC evaluations of metrics derived from Z‐spectra should thereby be taken with caution, as they might over‐extract information. This observation is in good agreement with previous reports about the inaccuracy of Lorentzian models in the fast exchange regime [32, 33, 34]. The efficacy of non‐localized models for quantification Z‐spectra has also been demonstrated previously, in form of heuristic polynomial models [35, 36].

We could also show that QUESP‐type experiments do not add information about very fast exchanging pools, as all $B_{1}$‐dependent terms vary only to a negligible amount. This implies that it is principally not possible to separate sufficiently fast exchanging pools with Z‐spectroscopy, independent from chosen saturation parameters.

In our phantom experiments, we used Glu and Glc as examples for pools in the intermediate and fast exchange regime [26, 27]. Conventional analysis techniques using multi‐pool Lorentzian fitting and MTR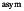 could successfully quantify the slower exchanging Glu ($R^{2}>95\%$), but Glc could not be quantified accurately when the Glu concentration increased ($R^{2}<45\%$). Similarly, the full analytical model also failed to quantify glucose accurately ($R^{2}<25\%$), although it does not assume any particular exchange regime. In contrast, the AB‐MT+n model could quantify fast‐exchanging Glc ($R^{2}>80\%$). Although explicitly considering a Glc pool in the AB‐MT+n model was beneficial to improve quantification results, the pool‐unspecific asymmetry parameter A reflected the Glc concentration best. It showed the least interference with the competing Glu concentration, although all exchanging pools additively contribute to A, by definition. This enforces the previous observation that asymmetry effects of fast‐exchanging pools are non‐localized. For non‐constant Glu concentration, quantification quality depended on the chosen saturation scheme, indicating that different weightings can be created through the $A(\omega_{1})$‐dependency.

### 5.2 Practical Application of the AB‐MT+n Model

The value of the AB‐MT+n model lies in its modular nature. It allows the model order n to be chosen based on the number of distinct observable off‐resonant effects rather than the number of theoretically contributing pools. The practical advantage of this could be shown in the GM seven‐pool simulation. Although all simulated pools contributed to the measured Z‐spectra in principle, they were not necessarily separable in practice. Due to the substantial spectral overlap of rNOE, amide, amine, guanidino, and hydroxyl contributions, together with the broad MT background, the information content of the spectra was lower than the nominal number of exchanging compartments would suggest. Accordingly, the BIC favored a reduced model order, indicating that the experimentally observable complexity is smaller than the underlying biophysical complexity. This effect became more pronounced at higher $B_{1}$, where broader saturation and stronger overlap further diminish the distinguishability of individual pools, such that at $B_{1}=4.0\;\mu$T the AB‐MT part alone was sufficient to accurately describe the spectra. These findings support the interpretation of AB‐MT+n as an effective model whose order should be selected according to spectral resolvability rather than assumed tissue composition. Selection of optimal model order remains a manual task yet, as no straightforward decision rule exists, but it may be automatized in future using machine learning approaches.

Additional advantages of the AB‐MT+n model include the feasibility of arbitrary MT line‐shapes [11], as well as explicit corrections of $B_{0}$‐, $B_{1}$‐ and $T_{1}$‐inhomogeneities, compared to implicit correction methods, requiring multiple‐$B_{1}$ acquisitions [37].

Taken together, these benefits enabled us to perform robust quantification of glucoCESL data in a mouse brain using the lowest‐order AB‐MT variant. Despite the absence of an explicit Glc pool in the model, glucose‐induced changes could be captured reproducibly through the asymmetry parameter A. The observed post‐infusion increase in A across large parts of the brain, together with a significantly positive whole‐brain mean ΔA, indicates that the AB‐MT component already provides a sufficiently sensitive effective description of the relevant off‐resonant glucose‐related contrast in vivo. At the same time, the model yields additional quantitative tissue contrasts through the A, B, and $f_{\text{MT}}$ maps. Importantly, these results demonstrate that accurate and biologically meaningful quantification does not necessarily require explicit modeling of all potentially contributing exchange pools. Instead, for acquisition schemes dominated by broad and overlapping contributions, a reduced effective model may be preferable because it preserves robustness and interpretability while avoiding unnecessary parameterization.

Alternative quantification approaches may utilize the line‐width of the DS, here reflected by the parameter B, in line with recent reports [38] which metrify the DS width for low saturation powers. The AB‐MT part may also be used as a baseline Z‐spectrum, analogous to existing background subtraction approaches [39, 40].

### 5.3 Assumptions and Limitations of the AB‐MT+n Framework

The AB‐MT+n framework was theoretically derived under the assumption of cw saturation and practical approximations to derive closed‐form analytical solutions to BM equations. These include sufficiently long saturation, an approximately mono‐exponential approach to steady state, small exchanging‐pool fractions $\left(f_{i}\ll 1\right)$, and negligible longitudinal relaxation of the exchanging pools. For strongly pulsed, transient, or otherwise non‐cw‐like acquisition schemes, its applicability should therefore be validated against numerical simulations or experimental calibration data.

The readout correction introduced in Equation (25) extends the practical signal model but does not remove these assumptions. It accounts for relaxation losses between saturation and readout as well as incomplete recovery between consecutively sampled offsets. However, the exchange‐dependent relaxation terms themselves remain based on the approximate cw theory.

A central limitation concerns the interpretation of fitted parameters. Explicit components should only be interpreted as pool‐specific when the corresponding off‐resonant contributions are sufficiently separable under the chosen acquisition conditions. This was illustrated by the additional $R_{\text{ex}}^{\text{off}}$‐based GM analysis: retained components at low and intermediate saturation powers corresponded to separable off‐resonant spectral contributions, most robustly rNOE and amide, whereas the guanidino‐matched component showed substantially larger deviations from the ground‐truth contribution. The guanidino pool therefore represents a borderline case, as its exchange rate is higher than for amide and rNOE and its spectral distance to nearby amine and hydroxyl resonances and on‐resonant effects is limited. Thus, a retained component should not automatically be interpreted as quantitatively reliable biochemical pool recovery. Information criteria such as the BIC can indicate the number of experimentally resolvable spectral degrees of freedom, but they do not identify the biochemical origin or quantitative reliability of retained components. The choice and interpretation of n should therefore also consider residual structure, parameter stability, expected resonance positions, acquisition settings, and the biological question of interest.

Further, although different MT line‐shapes can be incorporated into the AB‐MT+n framework, the present work was restricted to comparatively narrow CEST/CESL offset regimes around the water resonance; applications outside this regime would require dedicated validation.

Taken together, these limitations emphasize that AB‐MT+n should be used to extract the resolvable information contained in a given cw Z‐spectrum. Further improvements require complementary measurements that add independent information about exchange, relaxation, or more advanced analysis strategies.

### 5.4 Strategies to Overcome Current Limitations

The following strategies may help resolve information that is not identifiable from cw Z‐spectra using AB‐MT+n alone:

1. *Multi‐pH calibration of glucose exchange*: Zaiss et al. [26] demonstrated that using multi‐pH measurements of natural D‐glucose allows for calibration of exchange rates under physiological conditions, compared to multi‐$B_{1}$ measurements only.
2. *Prior information and multi‐contrast experiments*: Incorporating independently measured relaxation and exchange rates, chemical shifts as priors/constraints stabilizes fits and reduces crosstalk [41, 42, 43].
3. *AI, fingerprinting*: Dictionary‐based fingerprinting (MRF) and machine learning algorithms can map complex acquisition schedules to parameters with improved precision [44, 45, 46].
4. *Generalized Bloch modeling*: AB‐MT+n is limited to quantification of cw Z‐spectra. The generalized Bloch framework by Assländer et al. [47, 48] may offer additional insights when approaching the hard‐pulse limit.

## 6 Conclusion

The AB‐MT+n model provides an accurate, acquisition‐independent description of cw Z‐spectra and enables extraction of the maximally recoverable information content from Z‐spectra. The AB‐MT basis consists of five parameters that capture global contributions from DS, semi‐solid MT, and fast‐exchanging pools. This basis can be extended by the number of separably measurable pools, not leading to effective overfitting. Due to its modular structure and robustness, the AB‐MT+n framework is particularly suited for dynamic CEST/CESL acquisitions and for quantitative MRI approaches that integrate complementary readouts.

## Funding

This research was supported by the Deutsche Forschungsgemeinschaft (German Research Foundation) (Grant Nos. CRC1450‐431460824 and 493484898).

## Supporting information




**Figure S1:** Comparison of the effective water relaxation rate $R_{\text{eff}}$, the original MT relaxation rate contribution $R_{\text{ex}}^{\text{MT}}$, and the approximate MT expression used in the AB‐MT+n framework.
**Table S1:** Constant simulation parameters used for the four‐pool toy model and realistic gray matter model.
**Table S2:** Parameter bounds used for model fitting.

## Data Availability

The data that support the findings of this study are available from the corresponding author upon reasonable request.
