# Supplementary material for: Effective Modeling of Continuous Wave Z‐Spectra for Quantitative CEST and CESL MRI Across Exchange Regimes
Source: Magn Reson Med. 2026 Jul 30;96(5):2474–88. doi: 10.1002/mrm.70483 (PMC13527275; doi:10.1002/mrm.70483)
Supplement: Supplementary file 1 [file MRM-96-2474-s001.zip › Table_S1.pdf]

Table S1. Constant simulation parameters used for the four-pool toy model and realistic gray matter (GM) model.

Pool	f	T_1 [s]	T_2 [ms]	k [Hz]	$\delta\omega$ [ppm]
**Toy model**					
Water	1.0	1.0	100		
MT	0.1	1.0	0.01	40	-3.0
CE (a)	0.005	1.0	100	var.	1.5
CE (b)	0.005	1.0	100	var.	2.0
**GM**					
Water	1.0	1.2	69		
MT	0.0495	1.3	0.009	40	0.0
Amide	6.4865E-4	1.3	100	30	3.5
Amine	1.8018E-4	1.3	200	5500	3.0
Guanid.	1.8018E-4	1.3	170	1100	2.0
Hydroxyl	4.0541E-4	1.3	55	2000	0.9
rNOE	0.0045	1.3	5	20	-3.0
