# Supplementary material for: Effective Modeling of Continuous Wave Z‐Spectra for Quantitative CEST and CESL MRI Across Exchange Regimes
Source: Magn Reson Med. 2026 Jul 30;96(5):2474–88. doi: 10.1002/mrm.70483 (PMC13527275; doi:10.1002/mrm.70483)
Supplement: Supplementary file 1 [file MRM-96-2474-s001.zip › Table_S2.pdf]

Table S2. Parameter bounds for each model (AB-MT+n, full analytical, four-pool Lorentzian). The non-Lorentzian models share exchange pool parameter bounds.

Parameter	Minimum	Maximum
**AB-MT+n basis set**		
A	-0.1	0.1
B [ppm]	0.0	0.2
$R_{2,\mathrm{MT}}$ [ppm]	100.0	200.0
f_{MT}	0.001	0.100
$\delta\omega_{\mathrm{MT}}$ [ppm]	-4	-2
t_{delay} [ms]	12.5	100.0
$\sin\alpha_{\mathrm{eff}}$	0.6	1.0
**Full analytical basis set**		
$R_{1,\mathrm{w}}$ [ppm]	0.0005	0.02
$R_{2,\mathrm{w}}$ [ppm]	0.001	0.2
$R_{1,\mathrm{MT}}$ [ppm]	0.0005	0.02
$R_{2,\mathrm{MT}}$ [ppm]	100.0	200.0
k_{MT} [ppm]	0.01	1.0
f_{MT}	0.001	0.100
$\delta\omega_{\mathrm{MT}}$ [ppm]	-4	-2
**Glu pool parameters**		
f_{Glu}	0.00001	0.10000
k_{Glu} [ppm]	0.5	3.0
$\delta\omega_{\mathrm{Glu}}$ [ppm]	2.5	3.5
**Glc pool parameters**		
f_{Glc}	0.00001	0.10000
k_{Glc} [ppm]	1.0	5.0
$\delta\omega_{\mathrm{Glc}}$ [ppm]	1.5	2.5
**Four-pool Lorentzian**		
Z_{base}	0.3	1.0
Γ_{Water} [ppm]	1.0	100.0
a_{Water}	0.3	1.0
Γ_{MT} [ppm]	50.0	500.0
a_{MT}	0.02	0.4
$\delta\omega_{\mathrm{MT}}$ [ppm]	-4	-2
a_{Glu}	0.01	0.3
Γ_{Glu} [ppm]	1.0	20.0
$\delta\omega_{\mathrm{Glu}}$ [ppm]	2.5	3.5
a_{Glc}	0.01	0.3
Γ_{Glc} [ppm]	2.0	50.0
$\delta\omega_{\mathrm{Glc}}$ [ppm]	1.5	2.5
