# Supplementary material for: Effective Modeling of Continuous Wave Z‐Spectra for Quantitative CEST and CESL MRI Across Exchange Regimes
Source: Magn Reson Med. 2026 Jul 30;96(5):2474–88. doi: 10.1002/mrm.70483 (PMC13527275; doi:10.1002/mrm.70483)
Supplement: Supplementary file 1 [file MRM-96-2474-s001.zip › Figure_S1.pdf]

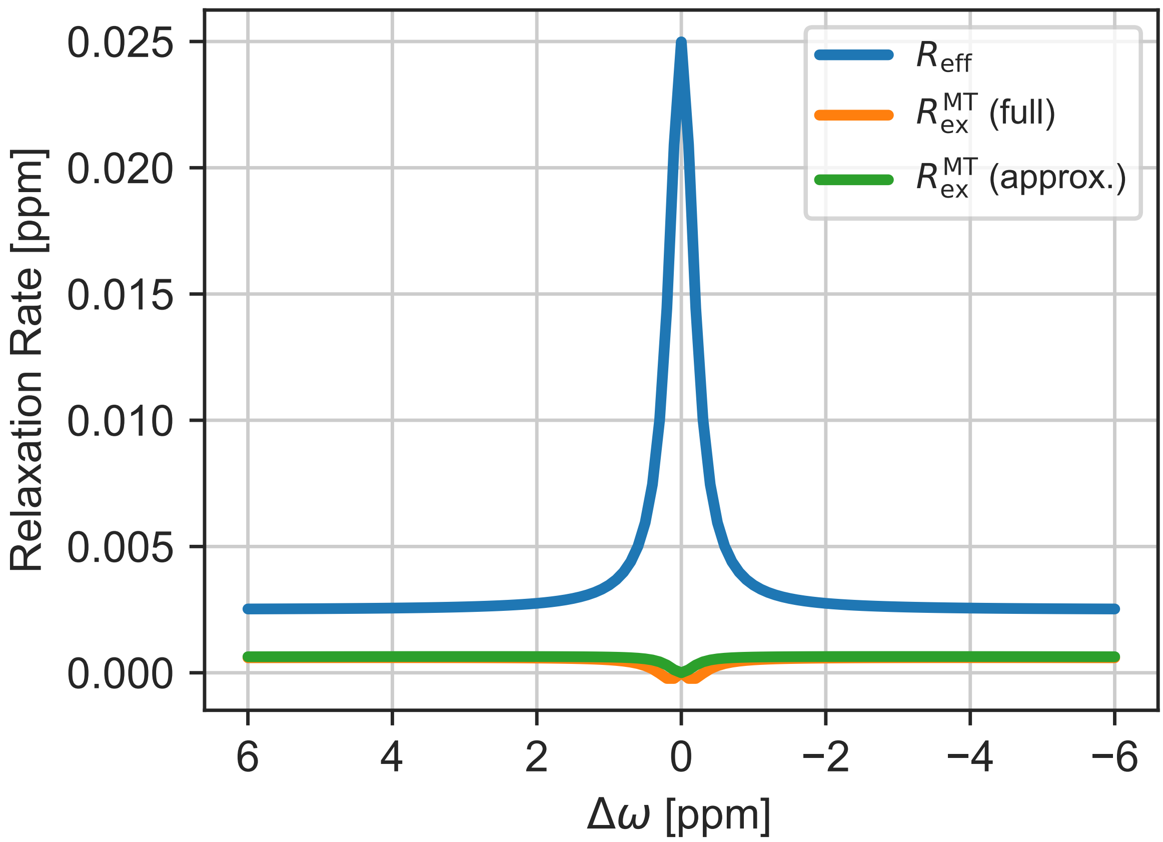

Figure S1. Comparison of the effective water relaxation rate R_{eff} (blue), the original MT relaxation rate contribution $R_{\mathrm{ex}}^{\mathrm{MT}}$ (orange), and the here introduced approximate formula (green). Parameter values were $B_0 = 9.4\,\mathrm{T}$, $B_1 = 2.0\,\mu\mathrm{T}$, $R_{1\mathrm{w}} = 1\,\mathrm{Hz}$, $R_{2\mathrm{w}} = 10\,\mathrm{Hz}$, $R_{1,\mathrm{MT}} = 1\,\mathrm{Hz}$, $R_{2,\mathrm{MT}} = 100\,\mathrm{kHz}$, $f_{\mathrm{MT}} = 0.1$, $k_{\mathrm{MT}} = 40\,\mathrm{Hz}$, $\delta\omega_{\mathrm{MT}} = -3\,\mathrm{ppm}$, with a Lorentzian MT absorption line-shape.
